# 2,2′-(Propane-1,3-di­yl)bis­(2*H*-indazole)

**DOI:** 10.1107/S1600536811029011

**Published:** 2011-07-30

**Authors:** Saúl Ovalle, Sylvain Bernès, Nancy Pérez Rodríguez, Perla Elizondo Martínez

**Affiliations:** a Laboratorio de Química Industrial, Centro de Laboratorios Especializados, Facultad de Ciencias Químicas, Universidad Autónoma de Nuevo León, Pedro de Alba S/N, 66451 San Nicolás de los Garza, NL, Mexico; b División de Estudios de Posgrado, Facultad de Ciencias Químicas, Universidad Autónoma de Nuevo León, Guerrero y Progreso S/N, Col. Treviño, 64570 Monterrey, NL, Mexico

## Abstract

The title mol­ecule, C_17_H_16_N_4_, is a bis-indazole crystallized in the rare 2*H*-tautomeric form. Indazole heterocycles are connected by a propane C_3_ chain, and the mol­ecule is placed on a general position, in contrast to the analogous compound with a central C_2_ ethane bridge, which was previously found to be placed on an inversion center in the same space group. In the title mol­ecule, indazole rings make a dihedral angle of 60.11 (7)°, and the bridging alkyl chain displays a *trans* conformation, resulting in a W-shaped mol­ecule. In the crystal, mol­ecules inter­act weakly through π–π contacts between inversion-related pyrazole rings, with a centroid–centroid separation of 3.746 (2) Å.

## Related literature

For the synthesis of 2*H*-indazoles, see: Wu *et al.* (2010 ▶). For studies of 1*H*←→2*H* tautomerism in indazoles, see: Alkorta & Elguero (2005 ▶); Yu *et al.* (2006 ▶). For 2*H*-indazole X-ray structures, see: Saczewski *et al.* (2001 ▶); Rodríguez de Barbarín *et al.* (2006 ▶); Ramos Silva *et al.* (2008 ▶); Hurtado *et al.* (2009 ▶); Zhou *et al.* (2010 ▶); Long *et al.* (2011 ▶).
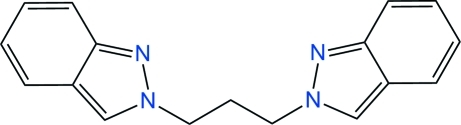


## Experimental

### 

#### Crystal data

C_17_H_16_N_4_
*M* *_r_* = 276.34
Orthorhombic, 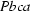

*a* = 8.182 (2) Å
*b* = 10.549 (4) Å
*c* = 34.179 (9) Å
*V* = 2950.1 (16) Å^3^
*Z* = 8
Mo *K*α radiation
μ = 0.08 mm^−1^
*T* = 298 K
0.60 × 0.40 × 0.18 mm

#### Data collection

Siemens P4 diffractometer
8195 measured reflections
2621 independent reflections
1607 reflections with *I* > 2σ(*I*)
*R*_int_ = 0.042
3 standard reflections every 97 reflections  intensity decay: 1.5%

#### Refinement

*R*[*F* ^2^ > 2σ(*F* ^2^)] = 0.049
*wR*(*F* ^2^) = 0.162
*S* = 1.10
2621 reflections
191 parameters
H-atom parameters constrained
Δρ_max_ = 0.20 e Å^−3^
Δρ_min_ = −0.21 e Å^−3^

### 

Data collection: *XSCANS* (Siemens, 1996 ▶); cell refinement: *XSCANS*; data reduction: *XSCANS*; program(s) used to solve structure: *SHELXS97* (Sheldrick, 2008 ▶); program(s) used to refine structure: *SHELXL97* (Sheldrick, 2008 ▶); molecular graphics: *SHELXTL* (Sheldrick, 2008 ▶) and *Mercury* (Macrae *et al.*, 2006 ▶); software used to prepare material for publication: *SHELXL97*.

## Supplementary Material

Crystal structure: contains datablock(s) I, global. DOI: 10.1107/S1600536811029011/aa2015sup1.cif

Structure factors: contains datablock(s) I. DOI: 10.1107/S1600536811029011/aa2015Isup2.hkl

Supplementary material file. DOI: 10.1107/S1600536811029011/aa2015Isup3.mol

Supplementary material file. DOI: 10.1107/S1600536811029011/aa2015Isup4.cml

Additional supplementary materials:  crystallographic information; 3D view; checkCIF report

## Figures and Tables

**Fig. 1.**
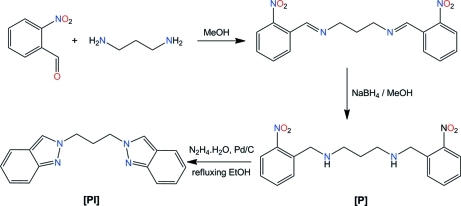
Synthetic route for the title compound. P is the key intermediate and PI is the title compound.

**Fig. 2.**
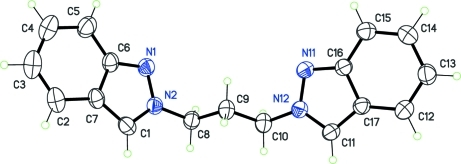
ORTEP-like view of the title molecule, with displacement ellipsoids at the 30% probability level for non-H atoms.

**Fig. 3.**
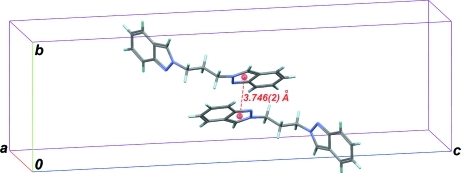
A part of the crystal structure representing two molecules related by inversion, which interact through a π–π contact involving pyrazole rings (dashed line).

## supplementary crystallographic information

### Comment

Although efficient synthetic routes for 2*H*-indazoles are available (*e.g*. Wu *et al.*, 2010) few molecules belonging to this family of heterocyclic compounds have been X-ray characterized up to now. This is due, in part, to the fact that 1*H* tautomer for indazoles is frequently more stable than the 2*H* tautomer, although the opposite situation may occur for some derivatives (Alkorta & Elguero, 2005; Yu *et al.*, 2006). On the other hand, in the case of N2-substituted indazoles, only the 2*H* tautomer is allowed. Tautomerism equilibrium study is important, since bioavailability and other pharmacological properties of indazoles may be dependent on such equilibria (*e.g*. Ramos Silva *et al.*, 2008). Indazole derivatives have been used, for instance, for their anti-inflammatory activity.

The title compound was prepared through a three steps procedure, the key step being the cyclization of a nitroamine derivative (compound P, see Fig. 1 and *Experimental*). The resulting bis-indazole consists of two 2*H*-indazole heterocycles connected by an alkyl C_3_ bridge (Fig. 2). The molecule lies in a general position, in space group *Pbca*. It is worth noting that the analogue compound with a central C_2_ bridge, for which we reported the X-ray structure (Rodríguez de Barbarín *et al.*, 2006), was found to crystallize in the same space group, although the molecule was placed on an inversion center. From this pair of structures now determined, we can propose the following general rule for *n*-alkyl bridged bis(2*H-*indazoles): even-alkyl compounds should be centrosymmetric, while odd-alkyl derivatives are expected to be non-centrosymmetric, as the title compound.

Molecular dimensions observed in the title compound compare well with those reported for other 2*H*-indazoles (Saczewski *et al.*, 2001; Rodríguez de Barbarín *et al.*, 2006; Hurtado *et al.*, 2009; Zhou *et al.*, 2010; Long *et al.*, 2011). Indazole rings make a dihedral angle of 60.11 (7)°. Torsion angles N2—C8—C9—C10 and C8—C9—C10—N12, -176.7 (2) and 173.5 (2)° respectively, characterize the *trans* conformation for the alkyl bridge, resulting in a W-shaped molecule. The crystal structure features π–π interactions of modest strength, between molecules related by inversion (Fig. 3). The separation between the centroid of the pyrazole ring N11/N12/C11/C17/C16 and the symmetry-related centroid at position -*x*, 1 - *y*, 1 - *z*, is 3.746 (2) Å.

### Experimental

The title ligand, PI, was obtained by a three steps reaction procedure (Fig. 1). The condensation between 1,3-diaminopropane and 2-nitrobenzaldehyde produced the corresponding imine. Selective reduction of imine bonds with sodium borohydride in methanol gave amine P, which was isolated. Then, 0.044 g of Pd/C was added to a solution of P (0.005 mol) in ethanol. This mixture was refluxed for 5.5 h, with addition of hydrazine monohydrate (0.110 mol) during the first 3 h. The mixture was filtered, distilled, and the organic phase was extracted. The product was purified by column chromatography with silica gel and ethyl acetate:hexane (2:1) as eluent. Suitable crystals were obtained by slow evaporation of an ethanol solution at 298 K. Mp 389.4–390 K; analysis found (calc. for C_17_H_16_N_4_): C 73.6 (73.9), H 5.8 (5.8), N 20.5% (20.3%); IR RTA: 3108 (CH Ar. ν_s_), 2950 (–CH_2_– ν_s_), 1625 (C=N Ar. δ_s_), 1514, 1467 (C=C Ar. ν_s_ and ν_as_). ^1^H NMR (300 MHz, CDCl_3_): δ, p.p.m.: 2.73 (2*H*, m, –CH_2_–), 4.41 (4*H*, t, N—CH_2_), 7.10 (2*H*, dd, Ar), 7.31 (2*H*, dd, Ar), 7.66 (2*H*, dd, Ar), 7.73 (2*H*, td, Ar), 7.95 (2*H*, s, NH).

### Refinement

All H atoms were placed in idealized positions and refined as riding to their parent C atoms, with bond lengths fixed to 0.97 (methylene CH_2_) or 0.93 Å (aromatic CH). Isotropic displacement parameters for H atoms were calculated as *U*_iso_(H) = 1.2 *U*_eq_(carrier atom).

### Crystal data

**Table:**

C_17_H_16_N_4_	*D*_x_ = 1.244 Mg m^−^^3^
*M**_r_* = 276.34	Melting point: 389 K
Orthorhombic, *P**b**c**a*	Mo *K*α radiation, λ = 0.71073 Å
Hall symbol: -P 2ac 2ab	Cell parameters from 77 reflections
*a* = 8.182 (2) Å	θ = 4.6–12.4°
*b* = 10.549 (4) Å	µ = 0.08 mm^−^^1^
*c* = 34.179 (9) Å	*T* = 298 K
*V* = 2950.1 (16) Å^3^	Prism, yellow
*Z* = 8	0.60 × 0.40 × 0.18 mm
*F*(000) = 1168	

### Data collection

**Table:**

Siemens P4 diffractometer	*R*_int_ = 0.042
Radiation source: fine-focus sealed tube	θ_max_ = 25.1°, θ_min_ = 2.4°
graphite	*h* = −9→8
ω scans	*k* = −12→12
8195 measured reflections	*l* = −40→40
2621 independent reflections	3 standard reflections every 97 reflections
1607 reflections with *I* > 2σ(*I*)	intensity decay: 1.5%

### Refinement

**Table:**

Refinement on *F*^2^	Secondary atom site location: difference Fourier map
Least-squares matrix: full	Hydrogen site location: inferred from neighbouring sites
*R*[*F*^2^ > 2σ(*F*^2^)] = 0.049	H-atom parameters constrained
*wR*(*F*^2^) = 0.162	*w* = 1/[σ^2^(*F*_o_^2^) + (0.0659*P*)^2^ + 0.9616*P*] where *P* = (*F*_o_^2^ + 2*F*_c_^2^)/3
*S* = 1.10	(Δ/σ)_max_ < 0.001
2621 reflections	Δρ_max_ = 0.20 e Å^−^^3^
191 parameters	Δρ_min_ = −0.21 e Å^−^^3^
0 restraints	Extinction correction: *SHELXL97* (Sheldrick, 2008), Fc^*^=kFc[1+0.001xFc^2^λ^3^/sin(2θ)]^-1/4^
0 constraints	Extinction coefficient: 0.0159 (17)
Primary atom site location: structure-invariant direct methods	

### Fractional atomic coordinates and isotropic or equivalent isotropic displacement parameters (Å^2^)

**Table:**

	*x*	*y*	*z*	*U*_iso_*/*U*_eq_	
N1	0.3628 (3)	0.67210 (17)	0.33910 (5)	0.0640 (6)	
N2	0.2422 (2)	0.75293 (17)	0.34924 (5)	0.0563 (5)	
C1	0.2608 (3)	0.8690 (2)	0.33476 (6)	0.0622 (7)	
H1A	0.1914	0.9378	0.3385	0.075*	
C2	0.4896 (4)	0.9559 (3)	0.29004 (7)	0.0773 (8)	
H2A	0.4529	1.0390	0.2876	0.093*	
C3	0.6261 (5)	0.9172 (3)	0.27178 (8)	0.0902 (10)	
H3A	0.6828	0.9744	0.2561	0.108*	
C4	0.6856 (4)	0.7935 (4)	0.27562 (9)	0.0986 (11)	
H4A	0.7815	0.7708	0.2628	0.118*	
C5	0.6062 (4)	0.7056 (3)	0.29769 (8)	0.0847 (9)	
H5A	0.6463	0.6235	0.3002	0.102*	
C6	0.4614 (3)	0.7431 (2)	0.31655 (6)	0.0610 (7)	
C7	0.4032 (3)	0.8675 (2)	0.31308 (6)	0.0604 (7)	
C8	0.1158 (3)	0.7097 (2)	0.37586 (6)	0.0639 (7)	
H8A	0.0822	0.6247	0.3686	0.077*	
H8B	0.0214	0.7649	0.3737	0.077*	
C9	0.1757 (3)	0.7094 (2)	0.41760 (7)	0.0646 (7)	
H9A	0.2737	0.6582	0.4195	0.078*	
H9B	0.2031	0.7952	0.4254	0.078*	
C10	0.0474 (3)	0.6575 (3)	0.44453 (6)	0.0671 (7)	
H10B	−0.0458	0.7143	0.4447	0.080*	
H10C	0.0109	0.5760	0.4348	0.080*	
N11	0.2399 (2)	0.56892 (18)	0.49101 (5)	0.0611 (6)	
N12	0.1075 (2)	0.64232 (18)	0.48438 (5)	0.0571 (5)	
C11	0.0399 (3)	0.6887 (2)	0.51674 (7)	0.0615 (7)	
H11B	−0.0516	0.7409	0.5181	0.074*	
C12	0.1239 (4)	0.6545 (2)	0.58912 (7)	0.0696 (7)	
H12B	0.0444	0.7040	0.6012	0.083*	
C13	0.2351 (3)	0.5902 (3)	0.61062 (7)	0.0699 (7)	
H13B	0.2306	0.5947	0.6378	0.084*	
C14	0.3567 (3)	0.5170 (2)	0.59287 (8)	0.0707 (7)	
H14C	0.4308	0.4737	0.6086	0.085*	
C15	0.3702 (3)	0.5070 (2)	0.55328 (7)	0.0683 (7)	
H15B	0.4538	0.4603	0.5418	0.082*	
C16	0.2530 (3)	0.5701 (2)	0.53037 (6)	0.0533 (6)	
C17	0.1312 (3)	0.6448 (2)	0.54797 (7)	0.0555 (6)	

### Atomic displacement parameters (Å^2^)

**Table:**

	*U*^11^	*U*^22^	*U*^33^	*U*^12^	*U*^13^	*U*^23^
N1	0.0772 (15)	0.0555 (11)	0.0593 (11)	0.0036 (11)	0.0102 (11)	0.0011 (9)
N2	0.0647 (13)	0.0543 (10)	0.0499 (10)	0.0009 (10)	0.0040 (10)	0.0007 (8)
C1	0.0780 (18)	0.0525 (13)	0.0560 (13)	0.0050 (12)	−0.0062 (14)	0.0031 (10)
C2	0.107 (2)	0.0695 (16)	0.0558 (14)	−0.0202 (17)	−0.0037 (16)	0.0056 (12)
C3	0.113 (3)	0.095 (2)	0.0633 (17)	−0.036 (2)	0.0138 (18)	0.0004 (15)
C4	0.100 (3)	0.118 (3)	0.0780 (19)	−0.011 (2)	0.0320 (18)	−0.0046 (19)
C5	0.095 (2)	0.0832 (19)	0.0754 (17)	0.0069 (18)	0.0252 (17)	−0.0047 (15)
C6	0.0760 (17)	0.0596 (14)	0.0474 (12)	−0.0041 (13)	0.0039 (12)	−0.0038 (10)
C7	0.0796 (18)	0.0570 (13)	0.0447 (12)	−0.0064 (13)	−0.0031 (12)	0.0003 (10)
C8	0.0633 (16)	0.0713 (15)	0.0572 (13)	−0.0018 (13)	0.0053 (12)	0.0041 (11)
C9	0.0662 (17)	0.0707 (15)	0.0570 (13)	−0.0090 (13)	0.0045 (12)	0.0051 (12)
C10	0.0665 (17)	0.0764 (16)	0.0583 (14)	−0.0086 (14)	0.0020 (13)	0.0044 (12)
N11	0.0601 (13)	0.0607 (12)	0.0626 (11)	0.0070 (10)	0.0088 (10)	0.0013 (9)
N12	0.0542 (12)	0.0595 (11)	0.0576 (11)	0.0007 (10)	0.0071 (10)	0.0047 (9)
C11	0.0608 (16)	0.0610 (14)	0.0628 (15)	0.0081 (12)	0.0103 (12)	0.0004 (11)
C12	0.0737 (18)	0.0719 (16)	0.0631 (15)	0.0076 (15)	0.0085 (14)	−0.0050 (12)
C13	0.0792 (19)	0.0731 (16)	0.0574 (13)	0.0005 (16)	−0.0023 (14)	0.0011 (12)
C14	0.0732 (19)	0.0667 (15)	0.0724 (16)	0.0059 (14)	−0.0063 (14)	0.0087 (13)
C15	0.0687 (17)	0.0639 (14)	0.0724 (16)	0.0098 (13)	0.0054 (14)	0.0012 (12)
C16	0.0535 (14)	0.0470 (11)	0.0593 (13)	−0.0014 (11)	0.0061 (11)	0.0023 (10)
C17	0.0570 (15)	0.0505 (12)	0.0590 (13)	0.0018 (11)	0.0072 (12)	0.0015 (10)

### Geometric parameters (Å, °)

**Table:**

N1—C6	1.343 (3)	C9—H9A	0.9700
N1—N2	1.350 (3)	C9—H9B	0.9700
N2—C1	1.329 (3)	C10—N12	1.457 (3)
N2—C8	1.451 (3)	C10—H10B	0.9700
C1—C7	1.381 (4)	C10—H10C	0.9700
C1—H1A	0.9300	N11—C16	1.350 (3)
C2—C3	1.343 (4)	N11—N12	1.351 (3)
C2—C7	1.410 (3)	N12—C11	1.330 (3)
C2—H2A	0.9300	C11—C17	1.383 (3)
C3—C4	1.400 (5)	C11—H11B	0.9300
C3—H3A	0.9300	C12—C13	1.353 (4)
C4—C5	1.360 (4)	C12—C17	1.411 (3)
C4—H4A	0.9300	C12—H12B	0.9300
C5—C6	1.406 (4)	C13—C14	1.398 (4)
C5—H5A	0.9300	C13—H13B	0.9300
C6—C7	1.401 (3)	C14—C15	1.362 (4)
C8—C9	1.509 (3)	C14—H14C	0.9300
C8—H8A	0.9700	C15—C16	1.405 (3)
C8—H8B	0.9700	C15—H15B	0.9300
C9—C10	1.499 (3)	C16—C17	1.406 (3)
			
C6—N1—N2	103.55 (18)	C10—C9—H9B	109.5
C1—N2—N1	113.7 (2)	C8—C9—H9B	109.5
C1—N2—C8	127.2 (2)	H9A—C9—H9B	108.1
N1—N2—C8	118.95 (18)	N12—C10—C9	112.2 (2)
N2—C1—C7	106.6 (2)	N12—C10—H10B	109.2
N2—C1—H1A	126.7	C9—C10—H10B	109.2
C7—C1—H1A	126.7	N12—C10—H10C	109.2
C3—C2—C7	118.4 (3)	C9—C10—H10C	109.2
C3—C2—H2A	120.8	H10B—C10—H10C	107.9
C7—C2—H2A	120.8	C16—N11—N12	103.05 (18)
C2—C3—C4	121.9 (3)	C11—N12—N11	113.89 (19)
C2—C3—H3A	119.0	C11—N12—C10	126.6 (2)
C4—C3—H3A	119.0	N11—N12—C10	119.37 (19)
C5—C4—C3	121.5 (3)	N12—C11—C17	107.1 (2)
C5—C4—H4A	119.3	N12—C11—H11B	126.5
C3—C4—H4A	119.3	C17—C11—H11B	126.5
C4—C5—C6	117.7 (3)	C13—C12—C17	118.5 (2)
C4—C5—H5A	121.1	C13—C12—H12B	120.8
C6—C5—H5A	121.1	C17—C12—H12B	120.8
N1—C6—C7	111.5 (2)	C12—C13—C14	121.4 (2)
N1—C6—C5	127.7 (2)	C12—C13—H13B	119.3
C7—C6—C5	120.7 (2)	C14—C13—H13B	119.3
C1—C7—C6	104.6 (2)	C15—C14—C13	122.1 (2)
C1—C7—C2	135.6 (3)	C15—C14—H14C	118.9
C6—C7—C2	119.8 (3)	C13—C14—H14C	118.9
N2—C8—C9	111.3 (2)	C14—C15—C16	117.5 (2)
N2—C8—H8A	109.4	C14—C15—H15B	121.2
C9—C8—H8A	109.4	C16—C15—H15B	121.2
N2—C8—H8B	109.4	N11—C16—C15	127.3 (2)
C9—C8—H8B	109.4	N11—C16—C17	112.0 (2)
H8A—C8—H8B	108.0	C15—C16—C17	120.7 (2)
C10—C9—C8	110.7 (2)	C11—C17—C16	103.9 (2)
C10—C9—H9A	109.5	C11—C17—C12	136.2 (2)
C8—C9—H9A	109.5	C16—C17—C12	119.8 (2)
			
C6—N1—N2—C1	−0.6 (2)	C8—C9—C10—N12	173.5 (2)
C6—N1—N2—C8	−176.64 (19)	C16—N11—N12—C11	0.5 (3)
N1—N2—C1—C7	0.3 (3)	C16—N11—N12—C10	−176.1 (2)
C8—N2—C1—C7	176.0 (2)	C9—C10—N12—C11	126.4 (3)
C7—C2—C3—C4	−1.2 (4)	C9—C10—N12—N11	−57.4 (3)
C2—C3—C4—C5	1.0 (5)	N11—N12—C11—C17	0.0 (3)
C3—C4—C5—C6	0.2 (5)	C10—N12—C11—C17	176.3 (2)
N2—N1—C6—C7	0.6 (3)	C17—C12—C13—C14	1.0 (4)
N2—N1—C6—C5	−179.3 (2)	C12—C13—C14—C15	0.3 (4)
C4—C5—C6—N1	178.9 (3)	C13—C14—C15—C16	−2.1 (4)
C4—C5—C6—C7	−1.0 (4)	N12—N11—C16—C15	179.2 (2)
N2—C1—C7—C6	0.1 (2)	N12—N11—C16—C17	−0.8 (2)
N2—C1—C7—C2	178.5 (3)	C14—C15—C16—N11	−177.3 (2)
N1—C6—C7—C1	−0.5 (3)	C14—C15—C16—C17	2.7 (4)
C5—C6—C7—C1	179.5 (2)	N12—C11—C17—C16	−0.5 (3)
N1—C6—C7—C2	−179.2 (2)	N12—C11—C17—C12	−177.6 (3)
C5—C6—C7—C2	0.8 (4)	N11—C16—C17—C11	0.8 (3)
C3—C2—C7—C1	−177.9 (3)	C15—C16—C17—C11	−179.2 (2)
C3—C2—C7—C6	0.4 (4)	N11—C16—C17—C12	178.5 (2)
C1—N2—C8—C9	−97.6 (3)	C15—C16—C17—C12	−1.5 (3)
N1—N2—C8—C9	77.9 (3)	C13—C12—C17—C11	176.4 (3)
N2—C8—C9—C10	−176.7 (2)	C13—C12—C17—C16	−0.4 (4)
